# Deep Learning-Driven Prediction of Mechanical Properties of 316L Stainless Steel Metallographic by Laser Powder Bed Fusion

**DOI:** 10.3390/mi15091167

**Published:** 2024-09-21

**Authors:** Zhizhou Zhang, Paul Mativenga, Wenhua Zhang, Shi-qing Huang

**Affiliations:** 1 School of Mechanics and Construction Engineering, Jinan University, Guangzhou 510632, China; zwhdwy@hnu.edu.cn; 2 Laser Processing Research Laboratory, School of Engineering, The University of Manchester, Manchester M13 9PL, UK; p.mativenga@manchester.ac.uk; 3 College of Packaging Engineering, Jinan University, Zhuhai 519070, China

**Keywords:** laser powder bed fusion, deep learning, mechanical properties prediction, machine learning visualisation, 316L stainless steel

## Abstract

This study developed a new metallography–property relationship neural network (MPR-Net) to predict the relationship between the microstructure and mechanical properties of 316L stainless steel built by laser powder bed fusion (LPBF). The accuracy R^2^ of MPR-Net was 0.96 and 0.91 for tensile strength and Vickers hardness predictions, respectively, based on optical metallurgy images. Feature visualisation methods, such as gradient-weighted class activation mapping (Grad-CAM) and clustering, were employed to interpret the abstract features within the MPR-Net, providing insights into the molten pool morphology and grain formation mechanisms during the LPBF process. Experimental results showed that the optimal process parameters—190 W laser power and 700 mm/s scanning speed—yielded a maximum tensile strength of 762.83 MPa and a Vickers hardness of 253.07 HV_0.2_ with nearly full densification (99.97%). The study marks the first application of a convolutional neural network (MPR-Net) to predict the mechanical properties of 316L stainless steel samples manufactured through laser powder bed fusion (LPBF) based on metallography. It innovatively employs techniques such as gradient-weighted class activation mapping (Grad-CAM), spatial coherence testing, and clustering to provide deeper insights into the workings of the machine learning model, enhancing the interpretability of complex neural network decisions in material science.

## 1. Introduction

Establishing a deterministic quantitative relationship between the structural characteristics and mechanical properties (e.g., strength, stiffness, Vickers hardness, and fatigue strength) of 3D-printed metals remains challenging from a continuum mechanics perspective. It has been essential to develop predictive methods for the mechanical properties of 3D-printed metals based on comprehensive mechanical performance tests and imaging data. Deep learning algorithms have been employed to uncover the physical mechanisms that influence these properties, providing valuable insights for optimising the performance of 3D-printed metals and refining the parameters of the 3D printing process.

Machine learning first made its mark in materials science in the 1990s when artificial neural networks (ANNs) were used to predict the macro-mechanical behaviour of fibre/matrix interface debonding in ceramic matrix composites [1]. Cetinel et al. [2], in 2002, used a three-layer perceptron neural network to predict the self-tempering temperature, mechanical properties, and tissue volume percentage of steel after heat treatment, based on the steel bar diameter and quenching time. In 2004, Guessasma et al. [3] applied a four-layer perceptron to analyse the microstructure of APS aluminium–titanium oxide coatings, revealing that the phase content of alumina and titania mainly depended on the spraying process parameters. Onal et al. [4], in 2010, used digital image analysis to study pore characteristics and employed a BP neural network to predict cement’s compressive strength, achieving a high correlation coefficient (R^2^ = 0.9971) between predicted and actual values. Li et al. [5], in 2011, used a fuzzy neural network to analyse the isothermal compression of the TC11 alloy, with prediction variances ranging from 0.62 to 3.92. The introduction of AlexNet [6] in 2012 brought convolutional neural networks (CNNs) into widespread use in image recognition, although their application in metallographic image analysis was still limited. In 2015, DeCost et al. [7] used support vector machines to classify microstructure images, while Li et al. [8], in 2017, estimated cement compressive strength using CNN-based microstructure images. Kondo et al. [9] employed CNNs to predict ceramic ion conductivity and identify features affecting material performance, with a correlation coefficient of R^2^ = 0.64. Later, DeCost et al. [10] used t-distributed stochastic neighborhood embedding (t-SNE) clustering for ultra-high carbon steel microstructure images, aiding deep learning models in distinguishing different metallographic morphologies. Lubbers et al. [11] showed that CNNs could synthesise material microstructure images and achieve effective dimensionality reduction.

Recently, Butler et al. [12] reviewed the potential of artificial intelligence to accelerate the design, synthesis, characterisation, and application of molecules and materials. Isayev et al. [13] developed a machine learning algorithm capable of predicting the properties of new materials, including metals, ceramics, and other crystals, and finding new applications for existing materials. In 2016, Nikolaev et al. [14,15] introduced ARES, the first autonomous material preparation machine that successfully synthesised single-molecule carbon nanotubes at the target growth rate. Ren et al. [16] used machine learning models trained on prior data and theoretical parameters to discover three new metallic glass compositions in the Co-V-Zr ternary system. Liu et al. [17] employed a backpropagation neural network to predict tensile strength, achieving a maximum absolute deviation of 67.32 MPa. They conducted a sensitivity analysis on input parameters and used the network to create contour maps guiding optimisation. Zhuo et al. [18] combined support vector machines with regression models and high-throughput density functional theory calculations to predict the Debye temperature, an indicator of photoluminescence quantum yield. In 2018, Azimi et al. [19] utilised a fully convolutional neural network to classify different microstructures of low-carbon steel with 93.94% accuracy, using pixel segmentation to identify microstructural areas automatically. Sanchez-Lengeling et al. [20] proposed an inverse design method for discovering materials with specific functions, leveraging generative adversarial networks (GANs) and reinforcement learning to generate molecules with desired properties.

Recent research showcased the application of machine learning (ML) and simulation technologies to enhance the properties and processes of additive manufacturing, with studies demonstrating ML’s ability to optimise parameters and improve material quality, such as 316L stainless steel and PLA materials. Additionally, advancements in ML for defect detection in laser-based additive manufacturing have significantly improved product reliability and quality, highlighting the potential of these technologies to transform industry standards and production efficiency. Eshkabilov et al. [21] focused on using machine learning to optimise process parameters in the laser powder bed fusion of 316L stainless steel, highlighting the ability to predict properties like density and mechanical strength through various algorithms. Similarly, Ji-Hye Park et al. [22] applied convolutional neural networks to assess the mechanical properties of ultrasonically treated PLA materials, offering insights into the improvements achieved through post-processing. Additionally, Kascak et al. [23] delved into the simulation of 316L stainless steel production, using tools to predict and mitigate potential errors and deformations in the manufacturing process, thus enhancing reliability and performance. Nasiri et al. [24] discussed the broader application of machine learning in predicting the mechanical behaviour of various additively manufactured parts, serving as a testament to the expanding role of artificial intelligence (AI) across different materials and methods. Wang et al. [25] explored the potential of machine learning in predicting the mechanical properties of Ti6Al4V alloys produced by laser powder bed fusion, with a focus on the influence of microstructural features. Integrating machine learning (ML) into laser-based additive manufacturing (LBAM) processes has sparked significant advancements in detecting and correcting defects, contributing to enhanced reliability and product quality. Fu et al. [26] provided a comprehensive review of ML algorithms used for defect detection in metal LBAM, covering various aspects including algorithm type, material specifics, defect categorisation, and dataset characteristics. Zhang et al. [27] comprehensively examined how machine learning enhances various aspects of 3D printing technology. The review focused on integrating supervised learning for process optimisation and surface quality control alongside reinforcement and deep learning for efficient path planning in multi-degree-of-freedom printing platforms. This research highlighted the transformative potential of machine learning in advancing additive manufacturing, making it more adaptable and efficient for high-end applications.

Deep learning has been instrumental in synthesising new materials, optimising material processes, classifying and identifying microstructures, and predicting material properties. However, there is still a gap in using convolutional neural networks, which excel in image processing, to predict the quantitative relationships between metallographic images and the mechanical properties of materials. Previous studies have often overlooked the internal learning characteristics of neural networks, particularly the mesostructured features encoded in network weights, and have failed to reveal how these features influence mechanical properties. Consequently, these studies lack the predictive mechanics and deeper understanding necessary for performance optimisation. This paper addresses these gaps by utilising various neural network models for accurate predictions and employing multiple visualisation methods to reveal the internal structural features learned by the networks.

## 2. Methods

### 2.1. Laser Powder Bed Fusion Process

In this study, the specimens were prepared by a laser powder bed fusion equipment model FS121M (Hunan Huashu High-Tech Co., Ltd., Changsha, China). We selected the laser power and scanning speed as the primary parameters. The laser power ranged from 140 to 190 W and the scanning speed from 600 to 900 mm/s were used to fabricate tensile test specimens on a 45 steel substrate. Other process parameters were kept constant. Based on the recommendations of the machine manufacturer for 316L stainless steel, the fixed parameters were as follows: a laser spot diameter of 130 μm, a line spacing of 90 μm, and a powder layer thickness of 30 μm. A layer rotation angle of 67° effectively inhibited the growth phenomenon of columnar crystals and increased the randomness of grain orientation in the melt pool. Alsalla et al. [28] explored how structural orientation affects the mechanical properties of SLM-fabricated 316L stainless steel samples, finding that specimens in a flat orientation exhibited the best mechanical properties. Thus, this orientation was adopted for the experiment. During the SLM process, to prevent the oxidation of the specimens and splattering of the powder, oxygen levels were kept below 5000 ppm. The entire manufacturing process took place in a nitrogen-filled protective chamber, with the pressure of the circulating gas system below 100 Pa. There was no post-processing heat treatment for the built specimens.

### 2.2. Mechanical Property Test

In this experiment, tensile specimens with a total length of 89.07 mm were printed on each substrate. The laser powers used were 190 W, 170 W, and 140 W, and the scanning speeds were 600 mm/s, 700 mm/s, 800 mm/s, and 900 mm/s. The metal tensile specimens were designed and formed according to the Reference [29]. The printed metal samples were cut using wire electrical discharge machining (EDM). For each set of process parameters, three tensile specimens with a thickness of 3 mm were cut. The tensile tests were conducted at room temperature using an MTS 810 material testing machine at a 2 mm/min loading speed. The VIC-3D™ system (Correlated Solutions, Inc., Columbia, SC, USA) equipped with two 4.1MPixel CMOSIS cameras was used to measure the strain. The DICM experimental procedure began with labelling each specimen and marking the gauge length, set to 31 mm according to the GB/T 228.1—2010 standard. The thickness and width of the specimens were measured to calculate the cross-sectional area, and paint was applied to mark the gauge points. Next, a high-speed camera was set up and connected to the data collection computer, with adjustments made to the exposure lights, camera position, aperture, and focus to ensure clear, reflection-free images of the specimens. The VIC-Snap 8 software was used for system calibration and image collection, with a capture interval of 500 ms. After the experiment, the collected images were analysed to obtain strain data, including transverse and longitudinal strains and the elongation of the specimens.

The Vickers hardness of each metallographic sample was measured using an HXD-1000TMSC/LCD Vickers hardness tester (Shanghai Taiming Optical Instrument Co., Ltd., Shanghai, China). A 200 g load was applied for an indentation time of 15 s. The Vickers hardness was measured three times on the cross-section of each sample along the tensile direction, and the average value was calculated. The surface of the samples was prepared to be flat and smooth, without oxidation or contaminants, especially grease. The samples were ground using water sandpaper from 600, 800, 1000, 1200, 1500, to 2000 grit, followed by polishing with W2.5 and W1.5 polishing paste. After polishing, the samples were cleaned in alcohol using ultrasonic cleaning and quickly dried. They were then wrapped in cotton for protection before testing. The thickness of the metallographic samples was 3 mm, which meets the minimum thickness requirement for the Vickers hardness measurement.

### 2.3. Structure of Neural Network and Training

**Figure 1:**
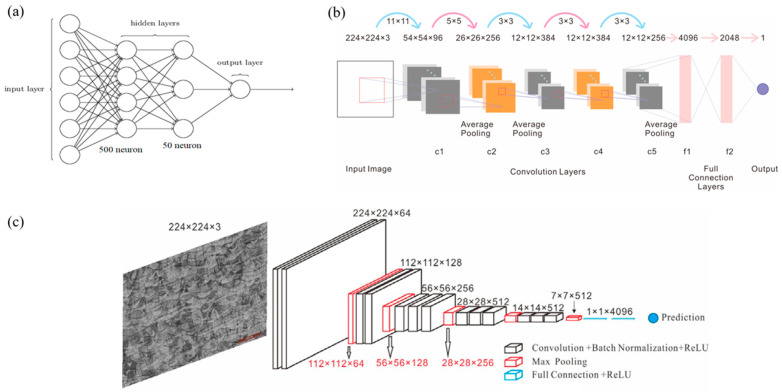
The algorithms used in this study were (**a**) the MLP structure, (**b**) the AlexNet revised structure for property prediction, and (**c**) the MPR-NET neural network structure.

The neural network structure used in this paper is shown in Figure 1. The metallographic image of 316L stainless steel used in this study had 224 × 224 pixels. As shown in Figure 1a, the structure of the MLP model in this study was that the six input vectors in the input layer were the porosity, the average area of the molten pool, the average perimeter of the molten pool boundary, the laser power, the scanning speed, and energy density. The first hidden layer had 500 neurons, the second hidden layer had 50 neurons, and the output layer had one, which was the predicted ultimate tensile strength. The activation function used in the MLP model was the sigmoid function. Figure 1a shows the architecture of the selected network. The use of this method to determine the relationship between structure and attributes depends, to a large extent, on the accuracy of the researcher’s hand-made features and skills. Therefore, this strategy lacks versatility for various microstructures, although humans can easily explain these characteristics.

The AlexNet architecture, a pioneering convolutional neural network (CNN) introduced by Alex Krizhevsky et al. [6], comprises eight layers designed for image classification tasks. As shown in Figure 1b, the revised network takes an input image of size 224 × 224 × 3, representing the width, height, and colour channels of the image. The architecture begins with five convolutional layers, each followed by a Rectified Linear Unit (ReLU) activation function, with the first two convolutional layers also followed by max-pooling layers. Following the convolutional layers, the network transitions to three fully connected layers, where the first two have 4096 neurons each, and the final layer contains 2048 neurons corresponding to the number of classes in the dataset. These fully connected layers also incorporate ReLU activation, with the final output being a mechanical property, such as the Vickers hardness and tensile strength. 

As shown in Figure 1c, the MPR-NET consisted of 13 convolutional layers plus three fully connected layers. All the convolution kernels of MPR-NET were 3 × 3 convolution filters. The activation function used by the convolutional layer and the fully connected layer of the MPR-NET model was the ReLU function. The architecture was characterised by its depth, consisting of 16 layers, 13 convolutional layers and three fully connected layers. The network takes an input image of size 224 × 224 × 3, representing the width, height, and colour channels, and passes it through a series of convolutional layers, each using 3 × 3 filters with a stride of 1 and padding of 1, preserving the spatial resolution of the feature maps. The convolutional layers are grouped into five blocks, each containing 2 or 3 convolutional layers followed by a max-pooling layer that reduces the spatial dimensions by a factor of 2. The first two blocks consist of two convolutional layers each, while the last three blocks consist of three convolutional layers each. As the network progresses through these layers, the number of filters doubles after each pooling layer, starting from 64 filters in the first block and reaching up to 512 filters in the last three blocks. Following the convolutional layers, the network transitions to three fully connected layers: the first two have 4096 neurons each, and the final layer contains one neuron corresponding to the property prediction value. ReLU activation functions follow these fully connected layers.

Each model was trained and validated with 80% of the dataset, and the remaining 20% of the dataset was for testing using the random permutation function presented by Numpy [30] for dataset splitting. The models were trained and validated using 12-fold cross validation. During the model training process, each batch was divided into 12 small mini-batches, 10% of the data in each minibatch was used for validation, and the remaining 90% of the data was used for training. The average value of root mean squared error (RMSE) loss of each output layer was used as the total loss function for gradient update. The coefficient of determination metrics R^2^ were used to evaluate the model’s performance by using a test dataset. All optimisations used Stochastic Gradient Descent (SGD) [31], where the objective function was the RMSE.

### 2.4. Dataset Processing and Labels

The laser 3D-printed 316L metallographic samples were manufactured with different laser powers and scanning speeds. The metallographic images were taken with an optical metallographic microscope, and the pixels of the metallographic image obtained were 2560 × 1920, and the resolution was 2.057 pixels/μm. In the metallographic diagram, the boundaries of the molten pool, pores, and over-corroded areas are depicted as dark areas. Therefore, the image contains the topological feature information of the 3D-printed metal sample. The number of metallographics in the dataset was 1200. The data of the metallographic image were enhanced. Each metallographic image was randomly cropped into 1000 × 1000 pixels, reduced to 224 × 224 pixels, and input into the convolutional neural network. The mechanical properties of this paper include the tensile strength and Vickers hardness, as shown in Table 1. The average tensile strength value was measured at room temperature by LPBF, which was used as the label. The average Vickers hardness values were used to label the metallographic images.

Batch normalisation [32] was used between the convolutional layer and the ReLu activate function, which can speed up the calculation process of convolutional neural networks. Batch normalisation is to force the distribution of the input value of any neuron in each layer of a neural network to a standard normal distribution with a mean value of 0 and a variance of 1 through a certain standardisation method. It is used to take the nonlinear function. The tensor distribution near the upper and lower lines of the value interval becomes a normal distribution so that the activation function input value falls in the area where the nonlinear function is more sensitive to the input, so that a small change in the input will cause a larger change in the activation function. That is done to make the gradient larger, to avoid the problem of gradient disappearance, and the larger gradient also means that the loss value converges faster and speeds up the training. The good thing about batch standardisation is that you can choose a larger initial learning rate, and adding a standardisation layer will increase the accuracy.

**Figure 2:**
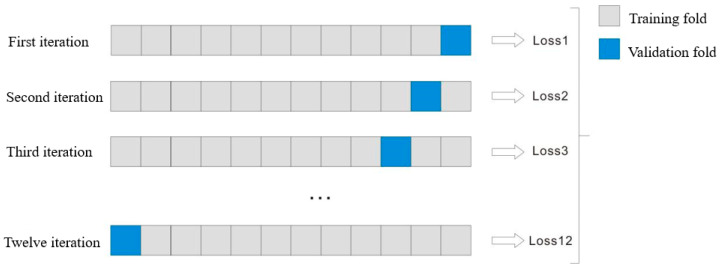
The schematic of the 12-fold cross validation method.

As shown in Figure 2, the use of leave-one-label-out cross validation to train the convolutional network was to prevent the neural network from overfitting. The training dataset had the tensile strength label (or the Vickers hardness label) set corresponding to 12 working conditions. During training, one label set was selected as the verification set, and the remaining 11 samples were used as the training set. For training, another label set in the training set was selected as the validation set for the next training, and the training was repeated many times. 

### 2.5. Metallographic Clustering

The characteristic of unsupervised learning is that there is only a large amount of data without corresponding labels, such as clustering. The dataset is automatically divided into clusters, and the samples in each cluster are similar. Dimensionality reduction projects high-dimensional data into low-dimensional space and retains information as much as possible. The role of dimensionality reduction is to visualise the data so that they can be observed and explored. Another role is to reduce the amount of machine learning calculations. In this study, the PCA and t-SNE algorithms were used for clustering.

Principal Component Analysis (PCA) is a commonly used data analysis method. The most significant feature of PCA technology is that there was no parameterisation. The PCA algorithm does not need to set hyperparameters such as the learning rate, iteration number, etc. The result of dimensionality reduction is only related to the data themselves. The lack of parameters to optimise learning in PCA means that the dimensionality reduction process cannot be fine-tuned, which may result in suboptimal outcomes. Next, we will introduce the t-SNE algorithm, which can perform iterative optimisation learning according to the set hyperparameters.

T-distributed Stochastic Neighbour Embedding (t-SNE) is a non-linear dimensionality reduction algorithm using a probabilistic form [33]. This algorithm converts the Euclidean distance between high-dimensional data into similarity probability and replaces the conditional probability in the t-SNE algorithm with joint probability, which alleviates the problem of mapping point crowding in the low-dimensional space of the original method. The t-SNE nonlinear dimensionality reduction algorithm finds patterns in the data by identifying observed clusters based on the similarity of data points with multiple features. It is essentially a dimensionality reduction and visualisation technology. In addition, the output of t-SNE can be used as the input features of other classification algorithms.

**Figure 3:**
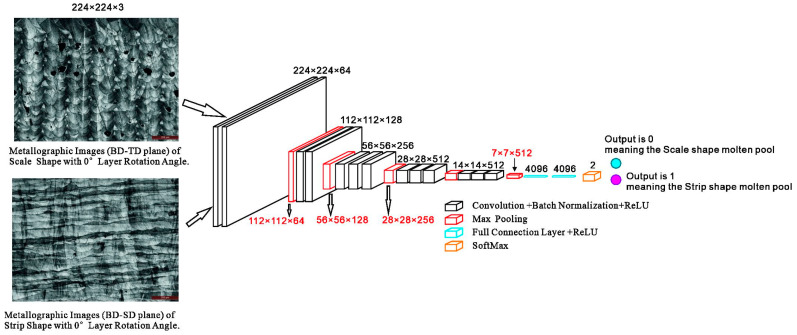
The two-class MPR-NET model of the scale-like side surface and the strip-like top surface of the specimen with an interlayer angle of 0°.

The structure of the MPR-NET model used for two classifications is shown in Figure 3. We will test the scale-shaped molten pool pictures and strip-shaped molten pool pictures in the test set and randomly cut 100 pictures for testing. The two-category MPR-NET model has an accuracy of 88.5% and a cross-entropy of 0.52.

The specific method of clustering fish scale and strip shape side was to crop 150 pictures in each label and convert the RGB channels to grayscale. The data were a tensor of 224 × 224 × 300. Note that the tensor form here must be distinguished from the dimension of the data. We deformed the matrix so that the 224 × 224 matrix becomes a column vector of 50,176 dimensions (rows). At this time, the data became a tensor of 50,176 × 300, which had 50,176 dimensions (rows) and 300 samples (columns). A 2 × 300 two-dimensional matrix was the output, where 2 was two-dimensional, representing the position coordinates of each picture in the 2D plane, and 300 represents the position of 300 picture clusters.

## 3. Result

### 3.1. Prediction of Tensile Strength

**Figure 4:**
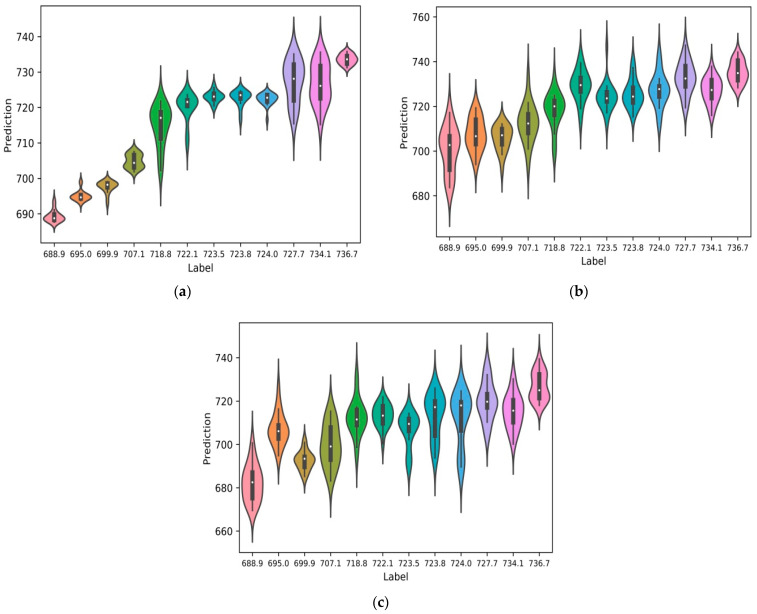
Predictive performance of neural networks trained with tensile strength label datasets. (**a**) Predicted value of tensile strength of MPR-NET. (**b**) Predicted value of tensile strength of AlexNet. (**c**) Predicted value of tensile strength of MLP.

As shown in Figure 4, the violin plot displays the data distribution and its probability density. The coefficient of determination R^2^ of the MPR-NET deep neural network was the highest at 0.96, followed by AlexNet at 0.81, and the lowest at MLP was only 0.45. This kind of chart combines the characteristics of box plots and density plots and is mainly used to show the distribution shape of data. The thick black bar in the middle represents the interquartile range, the thin black line extending from it represents the 95% confidence interval, the white point is the median, and the wave represents the frequency distribution. The predicted value range was wider when predicting 727.7 MPa and 734.1 MPa labels. The reason is that the working conditions were 170 W, 600 mm/s and 190 W, 700 mm/s, and there were a lot of pores at the bottom of the molten pool. Therefore, some metallographic diagrams have more pores. When using AlexNet to predict low-intensity tags, the larger prediction error was due to the uncertainty of the porosity when the energy density was low. By comparing the convolutional network with the traditional multi-layer perceptron (MLP), we can confirm that the convolutional network predicted the tensile strength from the original metallographic image more accurately than the MLP. Convolutional neural networks without any artificial image processing and input artificial features can be well trained, and their performance exceeds the traditional multi-layer perceptron MLP. After inspection, the low individual prediction values were caused by the selection of excessively corroded areas in the photo. When the predicted values for each working condition were averaged, the results closely aligned with the actual values, leading to a higher R^2^. There were partial misjudgements of individual pictures. In actual engineering, multiple images use the predicted average value as the standard to calculate the prediction accuracy and the coefficient of determination.

### 3.2. Prediction of Vickers Hardness 

**Figure 5:**
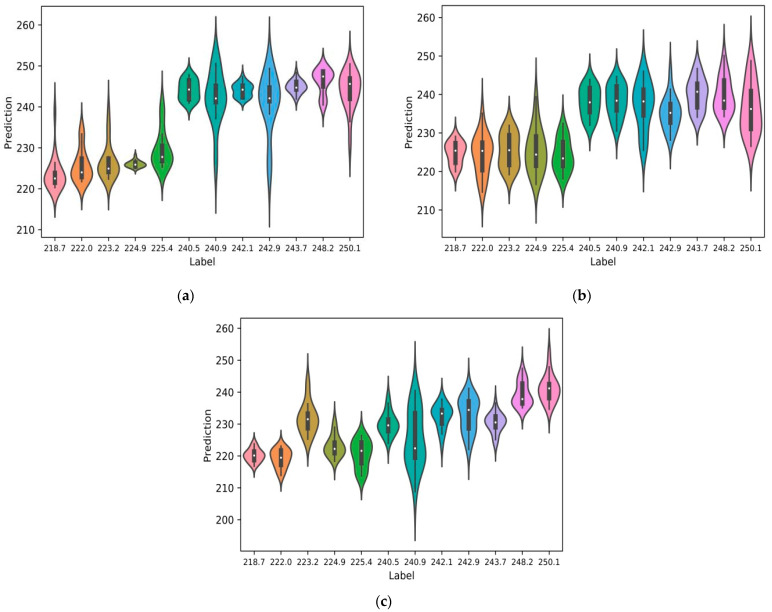
Predictive performance of neural networks trained with Vickers hardness label datasets. (**a**) Vickers hardness prediction value of MPR-NET. (**b**) Vickers hardness prediction value of AlexNet. (**c**) Vickers hardness prediction value of MLP.

As shown in Figure 5, Vickers hardness label datasets were used to train MPR-NET, AlexNet, and MLP models to predict Vickers hardness labels, and the average of these predicted values was used to calculate the coefficient of determination R^2^. The coefficient of determination R^2^ of the MPR-NET deep neural network was the highest at 0.91, followed by AlexNet at 0.70, and the lowest was MLP at 0.31. When MPR-NET predicted low-hardness labels, the predicted values of 240.9 HV_0.2_ and 242.9 HV_0.2_ had a larger range. The reason was that when the energy density was low, the partial porosity of some metallographic images in the test set was high.

### 3.3. Deep Learning Visualisation

The grade class activate map (Grad-CAM) [34,35] was used to obtain the activation map of the convolutional neural network. The formula for the gradient class activation map was derived as follows:

(1) $$w_{k}^{c}=\begin{array}{c} Global\;average\;pooling\\ \overbrace{\frac{1}{m}\sum_{i}\sum_{j}\left(\frac{\partial y^{c}}{\partial A_{ij}^{k}}\right)} \end{array}$$

*y^c^* is the predicted value of category *c*, *A^k^* is the feature map after convolution, $\frac{\partial y^{c}}{\partial A_{ij}^{k}}$ that is, the gradient between them. If global average pooling to this gradient matrix is performed, the importance weight of the *k*-th feature map $w_{k}^{c}$ is obtained. This weight represents the linearised weight of the *k*-th feature map $A_{ij}^{k}$ with respect to *y^c^*, and captures the “importance” of the feature map $A_{ij}^{k}$ of the target category *c*. We performed a weighted combination of forward activation maps and activated them through the ReLU function to obtain the $L_{Grad-CAM}^{c}$ gradient class activation map (Grad Class Activate Map, Grad-CAM).

(2) $$L_{Grad-CAM}^{c}=ReLU\underset{Linear\;weighting}{\underbrace{\left(\sum_{k}w_{k}^{c}A^{k}\right)}}$$

The ReLU activation function was applied to the linear combination of the feature maps because we were only interested in features that positively impact the category, that is, pixels whose value should be increased to increase the impact on the predicted value *y^c^*. Negative pixels belong to other categories in the image. As expected, without this ReLU function, the localisation mapping sometimes highlighted content other than the required classes, thereby reducing positioning performance. Generally speaking, *y^c^* does not need to be the class score generated by the image classification CNN. It can be any differentiable activation, including text and headings or answers to questions.

**Figure 6:**
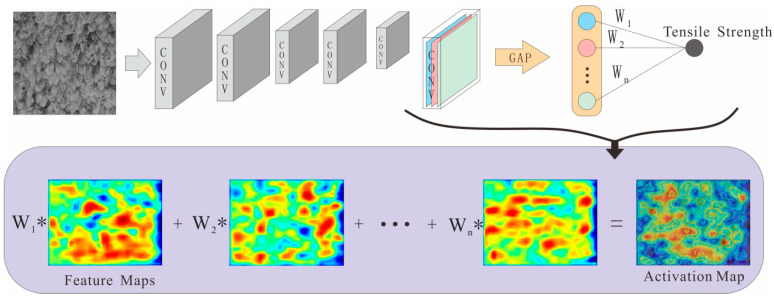
The schematic of the grade class activate map (Grad-CAM).

The principle of the gradient activation map is shown in Figure 6. The data were the feature map after the convolution of the last convolutional layer of the convolutional neural network. The feature map was added with the linear weight W to obtain the activation map, which can reflect the model-interested local areas in the image. The higher the activation value of this area, the greater the influence on the predicted value, representing the feature area with high tensile strength. The model used to visualise the internal features of the MPR-NET convolutional neural network was an MPR-NET model trained with tensile strength datasets, and the coefficient of determination R^2^ was 0.96.

**Figure 7:**
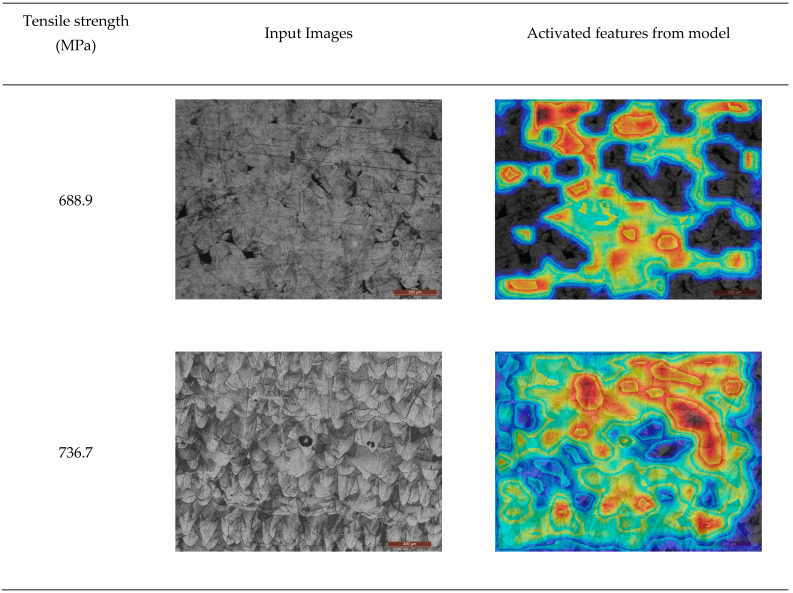
The grade class activate maps related to its tensile strength generated by the MPR-NET.

As shown in Figure 7, the metallographic image was imported into the trained MPR-NET. More Grad-CAMs related to different tensile strengths can be found in Supplementary Figure S1. The feature map of the convolutional layer’s last layer and the tensile strength’s linear weight were weighted. The area map of the convolutional neural network activation could be obtained. The activate maps showed that the neural network identifies the well-defined molten pool as contributing to tensile strength while the pore region remains inactive. The area of excessive corrosion has not been activated. Even if there were many local scratches in some pictures, the neural network could be activated, and it was worth noting that all the scale bars in the lower right corner of the input metallographic pictures were considered inactive because the scale bars were unrelated to the intensity. The metallographic diagram with high tensile strength has a large area of activated area, and the metallographic diagram with low tensile strength has a smaller activated area. There are many interlayer pores in the metallographic diagram with low tensile strength. The MPR-NET can automatically obtain statistics about the physical information of the pores and use this as a basis to judge the tensile strength.

**Figure 8:**
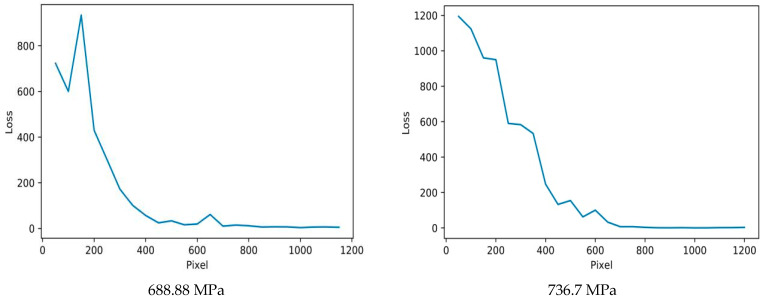
The MPR-NET neural network used the pixel versus mean absolute error curve of tensile strength dataset image prediction.

As shown in Supplementary Figure S2, metallographic images with different pixel resolutions are presented. The entire molten pool is visible at 600 × 600 or higher pixel resolution. The larger the pixel resolution, the more the molten pool can be seen. The metallographic images were randomly cropped at progressively higher pixel resolutions, beginning with 50 × 50 pixels and increasing in 50-pixel increments. Metallograph images with pixel dimensions ranging from 50 × 50 to 1200 × 1200 were fed into the MPR-NET neural network to compute the mean absolute error of its predictions. As shown in Figure 8 and Supplementary Figure S3, the ordinate represents the mean absolute error (MAE), and the abscissa represents the pixels of the cropped image. Cropping to obtain smaller images sometimes loses information about macro properties. For instance, images of dogs cannot be divided into separate images of the nose, ears, mouth, and tail, as these isolated parts no longer represent complete images of dogs. The representative volume element (RVE) is the smallest volume containing comparable images. In this study, a spatial coherence test was used to define the RVE. This essentially tests how well the spatial features in progressively larger image regions capture the material’s properties. As the image size increases, the microstructural features become more representative, and the predicted material properties stabilise. The smallest image size where this stabilisation occurs is the RVE, ensuring that this region is large enough to statistically represent the material without being unnecessarily large. In materials science, this process is essential to reduce computational complexity while ensuring accurate predictions of material behaviour based on microstructural features.

We randomly cut the metallographic pictures into pictures with different pixel sizes and imported them into the MPR-NET model for verification. The results show that the error gradually decreases as the image size increases. When the image pixel increases to around 600 × 600, the error no longer decreases but fluctuates in a small range. The resolution was 0.486 μm/pixel, and the size of the 600 × 600 pixel image was about 292 μm^2^, which was similar to the molten pool size. The length and width of the 600 × 600 pixel image were about 291 μm, which was larger than the maximum depth of 180 μm and width of 240 μm of the molten pool. It was proven that the convolutional neural network can identify the molten pool and use it as a typical representative unit (RVE).

### 3.4. Clustering Result

**Figure 9:**
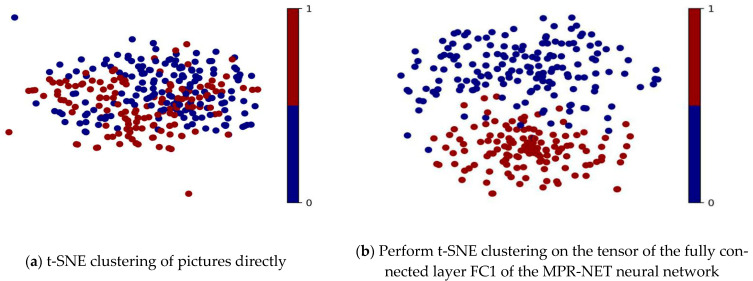
t-SNE was used to cluster (**a**) the metallographic optical images and (**b**) the tensor of the first fully connected layer.

As shown in Figure 9, red represents the bar shape, and blue represents the metallographic image of fish scales. In Figure 9a, the effect was not good when using t-SNE to cluster the metallographic image directly, and the enlarged image was shown in Supplementary Figure S4. In Figure 9b, the tensor of the first fully connected layer of the MPR-NET model was used to perform t-SNE clustering. This model can identify the two sides of a scale-shaped vertical surface and the strip-shaped parallel surface that distinguish the 0° interlayer rotation angle. The 4096-dimensional data of the first layer were exported and then the t-SNE algorithm was used to reduce the dimensionality to two-dimensional clustering. The enlarged view is shown in Supplementary Figure S5. In Figure 9b, the clustering effect was excellent, and the metallographic images of strip and fish scales were clearly separated into two types. As shown in Supplementary Figure S5, the interesting thing was that the bar-shaped pictures are darker under the entire clustering graph. Some metallographic images have relatively high brightness, which can eliminate the possibility of the neural network classifying light and dark based on the image and instead learn the texture of the melt pool.

**Figure 10:**
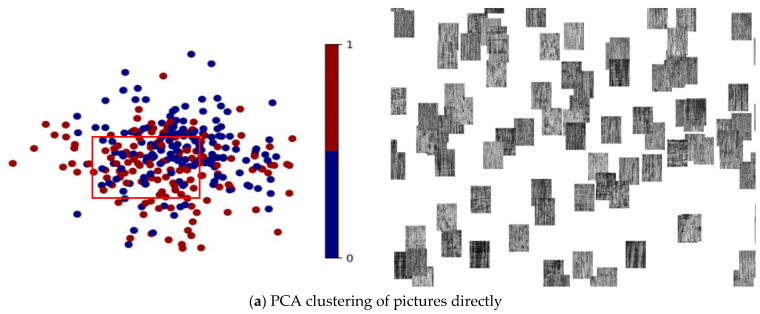

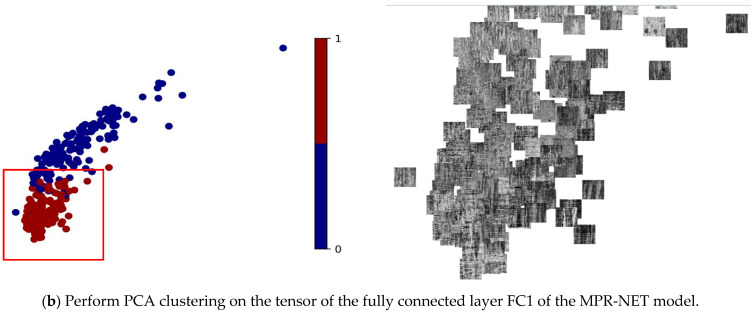
PCA was used to cluster (**a**) the metallographic optical images and (**b**) the tensor of the first fully connected layer of the MPR-NET model.

As shown in Figure 10, the blue dots represent the side of the fish scale, and the red dots represent the side of the strip. PCA clustering directly on the picture was not effective. The PCA clustering result of the tensor of the fully connected layer was good. The shape of PCA clustering by exporting the data of the fully connected layer was similar to a straight line, while t-SNE clustering was clustered into two semicircles. The shapes of clustering were related to the methods. PCA was linear dimensionality reduction. It was performed to maximise the data variance after dimensionality reduction. Therefore, when mapped to a 2D plane, it was divided into two halves along a straight line for clustering, and the data were projected. The difference was greatest above the straight line. The idea of t-SNE was to keep low-dimensional data with high-dimensional Euclidean distance information so it would be circular when dimensionality reduction was clustered to a 2D plane.

**Figure 11:**
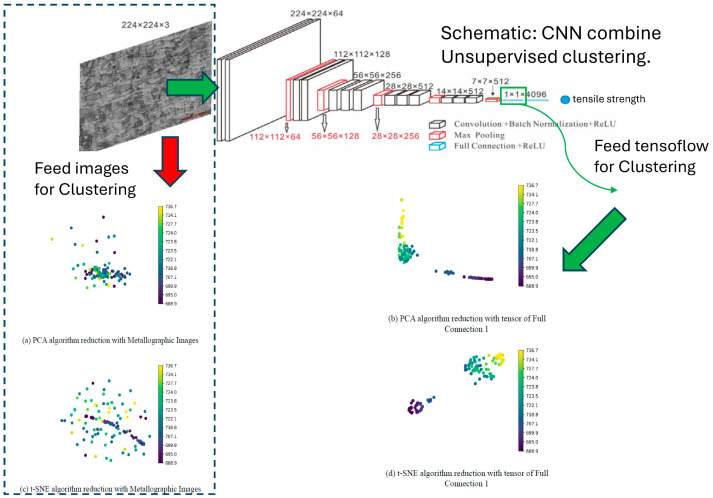
Schematic of feeding images for clustering directly and feeding tensor flow for clustering.

The Figure 11 compares direct image clustering using PCA and t-SNE and the clustering results obtained by feeding the tensor output from the first fully connected layer of the MPR-NET model into the same clustering algorithms. When PCA was applied directly to the metallographic images, the resulting clusters were mixed and indistinguishable, indicating poor clustering performance. Although t-SNE slightly enhances clustering by creating a linear cluster for images with lower tensile strength, images with higher tensile strength remain dispersed, highlighting the limitations of feeding images directly for clustering.

However, the effect was significantly better when the MPR-NET model processed the images and the tensor data from the first fully connected layer were used for PCA and t-SNE clustering. Most of the images with lower tensile strength were clustered together, which indicated that the neural network had effectively learned the features corresponding to the tensile strength. This improved clustering highlights the superiority of the neural network in extracting meaningful features, making it a powerful tool for analysing metallographic images in relation to their tensile strength.

**Figure 12:**
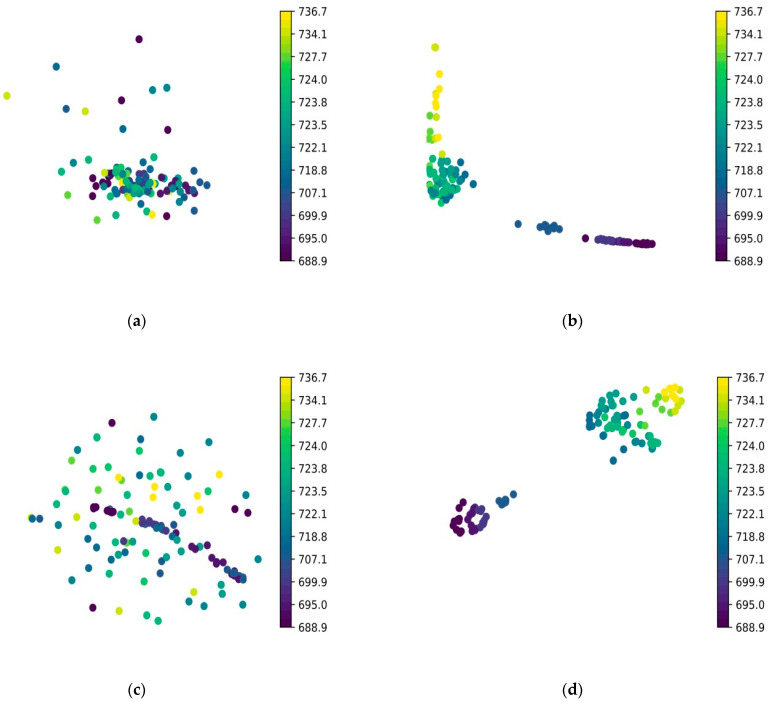
The PCA algorithm was used to cluster with inputs of (**a**) the metallographic optical images (interlayer angle of 67°) and (**b**) the tensor of the first fully connected layer of the MPR-NET neural network. The t-SNE algorithm was used to cluster with inputs of (**c**) the metallographic optical images (interlayer angle of 67°) and (**d**) the tensor of the first fully connected layer of the MPR-NET with tensile strength labels.

All the models used were trained MPR-NET models. As shown in Figure 12, metallographic images corresponding to different tensile strengths were clustered. In Figure 12a, PCA clustering was directly applied to the images, but the groups were intermixed and indistinguishable. In Figure 12c, t-SNE was used to classify the images, where those with lower tensile strength formed a linear cluster, but images with higher tensile strength remained scattered and unclustered. In Figure 12b, the metallographic images were input into the MPR-NET model, which was trained using tensile strength labels, and the output from the first layer of the fully connected network was used for PCA clustering. The clustering results were significantly better than those in Figure 12a, with most low-tensile-strength images clustered together, although some mixing between low and high-tensile-strength images still occurred. In Figure 12d, compared to Figure 12c, the clustering improved, with both low and high-tensile-strength images forming more distinct clusters, unlike the scattered distribution seen in Figure 12c. Overall, the clustering performance was less effective for the side-view metallographic images with a 67° rotation angle, likely due to the variability in weld pool size and less distinct texture features than the 0° rotation angle.

**Figure 13:**
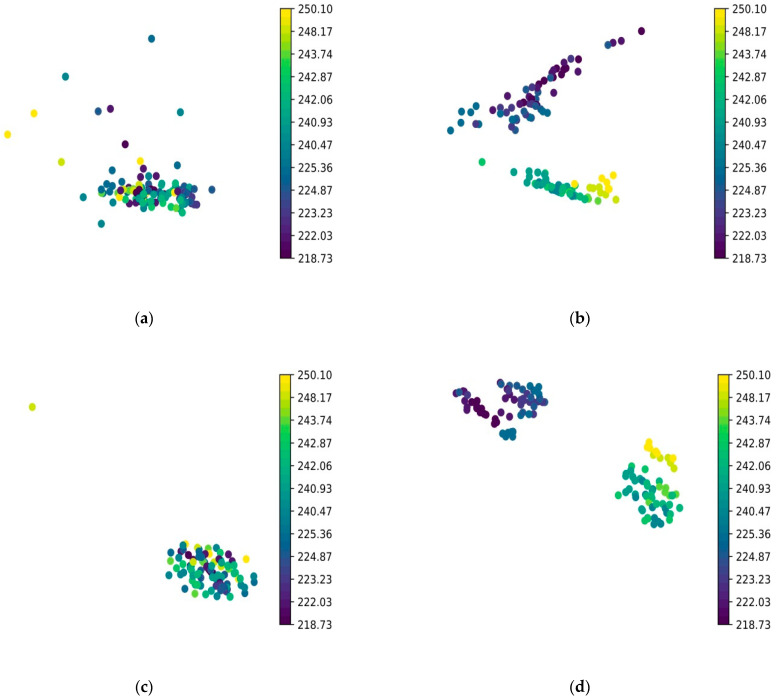
The metallographic images of Vickers hardness label (interlayer angle of 67°) was used for clustering. (**a**) PCA was fed directly by the metallographic optical images. (**b**) PCA clustering was performed on the tensor of the fully connected layer FC1 of the MPR-NET neural network that predicts Vickers hardness. (**c**) t-SNE was fed directly by the metallographic optical images. (**d**) t-SNE clustering was performed on the tensor of the fully connected layer FC1 of the MPR-NET neural network that predicts Vickers hardness.

Figure 13a,c shows that PCA and t-SNE clustering applied directly to the metallographic images yielded poor results. The metallography images with different Vickers hardness labels could not be grouped into distinct clusters.

As shown in Figure 13b, the metallographic images (the inter-layer rotation angle of 67°) were imported into the trained MPR-NET neural network for forward propagation, the 4096-dimensional data of the fully connected layer of the first layer were exported, and the PCA was reduced. From the 2D dimension, it can be seen that the metallographic diagrams of low hardness and high hardness were divided into two categories. The metallographic diagrams of some low-hardness labels were mixed and could not be dispersed because the metallographic diagrams of low hardness had similar texture characteristics, so it was not easy to distinguish. The same was true for high hardness. As shown in Figure 13d, the metallographic image with the interlayer rotation angle of 67° was imported into the trained MPR-NET neural network for forward propagation. The 4096-dimensional data of the fully connected layer of the first layer were exported, and after t- SNE dimensionality reduction, the metallographic maps were clustered into three categories; the metallographic maps with the 250.10 HV_0.2_ label were clustered into one category, and the metallographic maps with high hardness from 243.74 HV_0.2_ to 240.47 HV_0.2_ were clustered into one. Class 225.36 HV_0.2_ to 218.73 HV_0.2_ low-hardness metallographic diagrams were grouped into one category.

## 4. Conclusions and Outlooks

This study demonstrates the successful application of deep learning techniques, particularly the MPR-NET model, to predict the mechanical properties of 316L stainless steel produced by laser powder bed fusion (LPBF). The MPR-NET model was used to study the relationship between the microscopic morphology and mechanical properties of the built 316L specimens. The following main conclusions were drawn: The MPR-NET model achieved high accuracy in predicting both tensile strength (R^2^ = 0.96) and Vickers hardness (R^2^ = 0.91) based on metallographic images. The effect of PCA and t-SNE clustering on metallographic images through MPR-NET model dimensionality reduction was better than that of direct PCA and t-SNE clustering, indicating that the convolutional neural network learned the main features of the picture. By observing the activation area map of the convolutional neural network, it can be recognised that the area where the neural network learned to activate was a clear molten pool area, while the pores and excessively corroded areas were not activated. The higher the tensile strength, the larger the area that was activated. The convolutional network can count the physical information about the pores and use it to judge the tensile strength. Through the input of images of different sizes, the input pixels were more than 600 × 600 pictures and the accuracy of the convolutional neural network was high and stable, which proves that the convolutional neural network can identify the molten pool and regard it as a typical representative unit. Advanced feature visualisation techniques, such as Grad-CAM, provided valuable insights into the physical mechanisms, including molten pool morphology and grain formation, which govern these properties. The study further highlights the advantages of integrating convolutional neural networks (CNNs) for analysing metallographic images, which outperformed traditional methods like PCA and t-SNE in clustering and predicting material properties. This approach enhances our understanding of the relationship between microstructure and mechanical performance and paves the way for optimising LPBF process parameters using artificial intelligence.

There are several promising directions for further exploration in using artificial intelligence to optimise LPBF process parameters and enhance the performance of metal materials. First, the approach of predicting material properties using metallographic images can be extended beyond metals to other materials, provided photomicrographs and relevant characteristics like compressive strength, corrosion resistance, or electrical conductivity are available. This study opened the door to predicting a broader range of material properties. Additionally, advancing neural network visualisation techniques could offer deeper insights into how internal features influence material performance, enabling a better understanding of the physical properties learned by the network and how microstructures affect these properties. Furthermore, future research could focus on functionally graded materials (FGMs) produced by 3D printing, where AI could assist in predicting and optimising gradient process parameters to meet specific operational requirements. Finally, integrating finite element simulation with AI could provide a rich dataset for machine learning to uncover the evolution of material morphology and structure and crack propagation under thermal stress, thereby enhancing our predictive capabilities in this field.

## Figures and Tables

**Table 1:** Mechanical property labels for 12 working conditions.

Laser Power (W)	Scanning Speed (mm/s)	Energy Density (J/mm^3^)	Tensile Strength (MPa)	Vickers Hardness (HV_0.2_)
190	600	117.28	736.72 ± 16.08	242.06 ± 8.22
190	700	100.53	734.10 ± 14.93	250.10 ± 3.20
190	800	87.96	724.01 ± 3.83	242.87 ± 5.12
190	900	78.19	722.10 ± 3.68	240.47 ± 3.71
170	600	104.94	727.70 ± 9.81	248.17 ± 4.96
170	700	89.95	723.81 ± 3.02	243.74 ± 0.67
170	800	78.70	723.53 ± 5.81	240.93 ± 2.89
170	900	69.96	718.80 ± 3.84	222.03 ± 5.61
140	600	86.42	707.10 ± 8.89	224.87 ± 1.58
140	700	74.07	699.88 ± 16.41	218.73 ± 5.02
140	800	64.81	695.00 ± 18.00	223.23 ± 4.19
140	900	57.61	688.88 ± 15.27	225.36 ± 4.74

## Data Availability

Data will be made available on request.

## Supplementary Materials

The following supporting information can be downloaded at: https://www.mdpi.com/article/10.3390/mi15091167/s1, Figure S1: The grade class activate maps were generated by the MPR-NET with its tensile strength labels.; Figure S2: Metallograph images inputs with pixel dimensions ranging from 200 × 200 to 1100 × 1100 for MPR-NET.; Figure S3: The pixel versus mean absolute error (MAE) curve of tensile strength dataset image prediction via the MPR-NET model.; Figure S4: The clustering result of using t-SNE clustering on the images directly.; Figure S5: The t-SNE clustering result by feeding the tensor of the first fully connected layer of the MPR-NET.

## References

[1] Rao H.S., Mukherjee A. (1996). Artificial neural networks for predicting the macromechanical behaviour of ceramic-matrix composites. Comput. Mater. Sci..

[2] Cetinel H., Ozyigit H.A., Ozsoyeller L. (2002). Artificial neural networks modeling of mechanical property and microstructure evolution in the Tempcore process. Comput. Struct..

[3] Guessasma S., Coddet C. (2004). Microstructure of APS alumina-titania coatings analysed using artificial neural network. Acta Mater..

[4] Onal O., Ozturk A.U. (2010). Artificial neural network application on microstructure-compressive strength relationship of cement mortar. Adv. Eng. Softw..

[5] Li M.Q., Zhang X.Y. (2011). Modeling of the microstructure variables in the isothermal compression of TC11 alloy using fuzzy neural networks. Mater. Sci. Eng. A.

[6] Krizhevsky A., Sutskever I., Hinton G.E. (2017). ImageNet Classification with Deep Convolutional Neural Networks. Commun. ACM.

[7] DeCost B.L., Holm E.A. (2015). A computer vision approach for automated analysis and classification of microstructural image data. Comput. Mater. Sci..

[8] Li M.H., Wang L., Yang B., Zhang L.L., Liu Y. (2017). Estimating Cement Compressive Strength from Microstructure Images Using Convolutional Neural Network. Proceedings of the 2017 IEEE Symposium Series on Computational Intelligence (SSCI).

[9] Kondo R., Yamakawa S., Masuoka Y., Tajima S., Asahi R. (2017). Microstructure recognition using convolutional neural networks for prediction of ionic conductivity in ceramics. Acta Mater..

[10] DeCost B.L., Francis T., Holm E.A. (2017). Exploring the microstructure manifold: Image texture representations applied to ultrahigh carbon steel microstructures. Acta Mater..

[11] Lubbers N., Lookman T., Barros K. (2017). Inferring low-dimensional microstructure representations using convolutional neural networks. Phys. Rev. E.

[12] Butler K.T., Davies D.W., Cartwright H., Isayev O., Walsh A. (2018). Machine learning for molecular and materials science. Nature.

[13] Isayev O., Oses C., Toher C., Gossett E., Curtarolo S., Tropsha A. (2017). Universal fragment descriptors for predicting properties of inorganic crystals. Nat. Commun..

[14] Nikolaev P., Hooper D., Webber F., Rao R., Decker K., Krein M., Poleski J., Barto R., Maruyama B. (2016). Autonomy in materials research: A case study in carbon nanotube growth. Npj. Comput. Mater..

[15] Nikolaev P., Hooper D., Perea-Lopez N., Terrones M., Maruyama B. (2014). Discovery of Wall-Selective Carbon Nanotube Growth Conditions via Automated Experimentation. ACS Nano.

[16] Ren F., Ward L., Williams T., Laws K.J., Wolverton C., Hattrick-Simpers J., Mehta A. (2018). Accelerated discovery of metallic glasses through iteration of machine learning and high-throughput experiments. Sci. Adv..

[17] Liu G.X., Jia L.N., Kong B., Feng S.B., Zhang H.R., Zhang H. (2017). Artificial neural network application to microstructure design of Nb-Si alloy to improve ultimate tensile strength. Mater. Sci. Eng. A-Struct. Mater. Prop. Microstruct. Process..

[18] Zhuo Y., Tehrani A.M., Oliynyk A.O., Duke A.C., Brgoch J. (2018). Identifying an efficient, thermally robust inorganic phosphor host via machine learning. Nat. Commun..

[19] Azimi S.M., Britz D., Engstler M., Fritz M., Mucklich F. (2018). Advanced Steel Microstructural Classification by Deep Learning Methods. Sci. Rep..

[20] Sanchez-Lengeling B., Aspuru-Guzik A. (2018). Inverse molecular design using machine learning: Generative models for matter engineering. Science.

[21] Eshkabilov S., Ara I., Azarmi F. (2022). A comprehensive investigation on application of machine learning for optimisation of process parameters of laser powder bed fusion-processed 316L stainless steel. Int. J. Adv. Manuf. Technol..

[22] Park J.-H., Kim S.-H., Park J.-Y., Kim S.-G., Lee Y.-J., Kim J.-H. (2024). Prediction of Microstructure and Mechanical Properties of Ultrasonically Treated PLA Materials Using Convolutional Neural Networks. Int. J. Precis. Eng. Manuf..

[23] Kascak L., Varga J., Bidulska J., Bidulsky R. (2023). Simulation of 316L Stainless Steel Produced the Laser Powder Bed Fusion Process. Materials.

[24] Nasiri S., Khosravani M.R. (2021). Machine learning in predicting mechanical behavior of additively manufactured parts. J. Mater. Res. Technol..

[25] Wang H., Li B., Zhang W., Xuan F. (2024). Microstructural feature-driven machine learning for predicting mechanical tensile strength of laser powder bed fusion (L-PBF) additively manufactured Ti6Al4V alloy. Eng. Fract. Mech..

[26] Fu Y., Downey A.R.J., Yuan L., Zhang T., Pratt A., Balogun Y. (2022). Machine learning algorithms for defect detection in metal laser-based additive manufacturing: A review. J. Manuf. Process..

[27] Zhang X., Chu D., Zhao X., Gao C., Lu L., He Y., Bai W. (2024). Machine learning-driven 3D printing: A review. Appl. Mater. Today.

[28] Alsalla H.H., Smith C., Hao L. (2018). Effect of build orientation on the surface quality, microstructure and mechanical properties of selective laser melting 316L stainless steel. Rapid Prototyping J..

[29] (2010). Metallic Materials—Tensile Testing.

[30] Harris C.R., Millman K.J., Van Der Walt S.J., Gommers R., Virtanen P., Cournapeau D., Wieser E., Taylor J., Berg S., Smith N.J. (2020). Array programming with NumPy. Nature.

[31] Wang L., Yang Y., Min R., Chakradhar S. (2017). Accelerating deep neural network training with inconsistent stochastic gradient descent. Neural Netw..

[32] Ioffe S., Szegedy C. (2015). Batch Normalisation: Accelerating Deep Network Training by Reducing Internal Covariate Shift. arXiv.

[33] van der Maaten L., Hinton G. (2008). Visualizing Data using t-SNE. J. Mach. Learn. Res..

[34] Zhou B., Khosla A., Lapedriza A., Oliva A., Torralba A. (2016). Learning Deep Features for Discriminative Localization. Proceedings of the 2016 Ieee Conference on Computer Vision and Pattern Recognition.

[35] Selvaraju R.R., Cogswell M., Das A., Vedantam R., Parikh D., Batra D. (2017). Grad-CAM: Visual Explanations from Deep Networks via Gradient-based Localization. Proceedings of the 2017 IEEE International Conference on Computer Vision.

