# Supplementary material for: Deep Learning-Driven Prediction of Mechanical Properties of 316L Stainless Steel Metallographic by Laser Powder Bed Fusion
Source: Micromachines (Basel). 2024 Sep 21;15(9):1167. doi: 10.3390/mi15091167 (PMC11434083; doi:10.3390/mi15091167)
Supplement: Supplementary file 1 [file micromachines-15-01167-s001.zip › micromachines-3192412-supplementary.pdf]

Supplementary Information

Tensile strength (Mpa)	Input images	Activated features of high strength
688.9	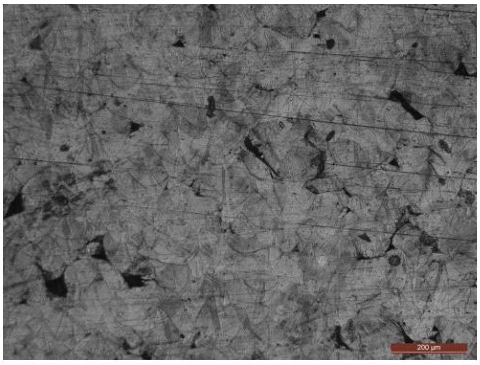	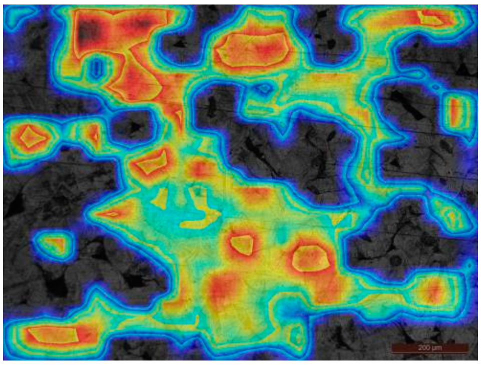
695.0	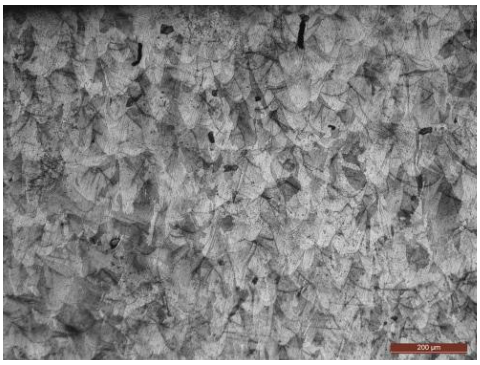	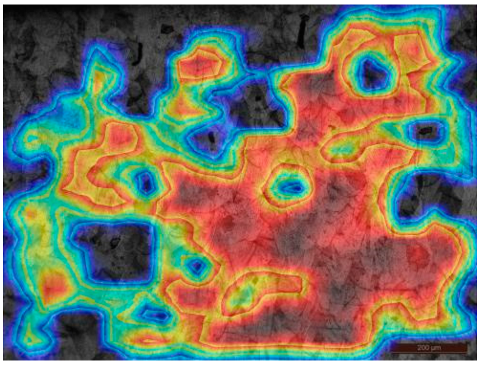
699.9	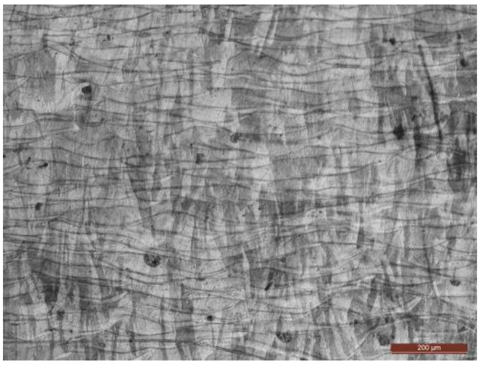	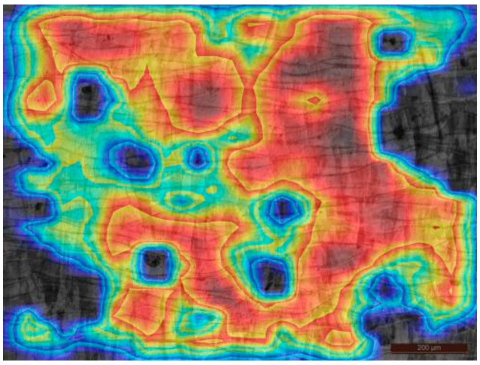

707.1

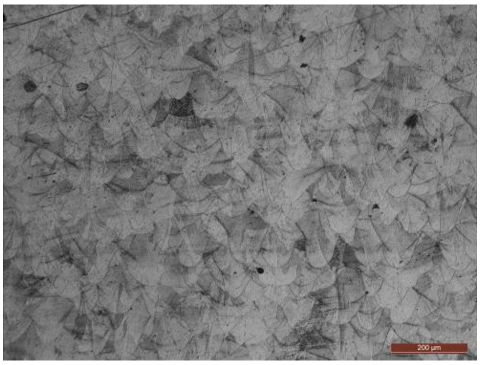

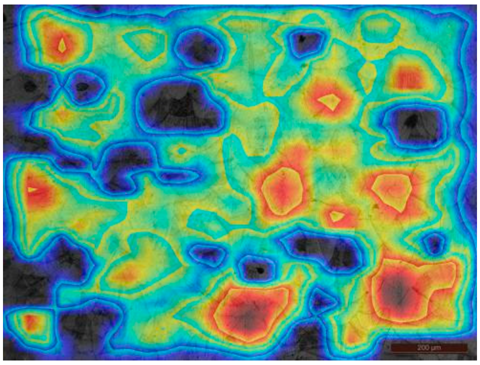

718.8

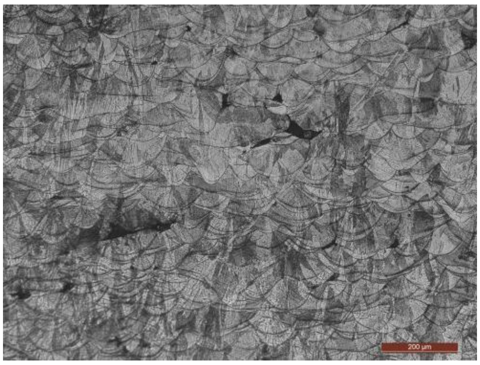

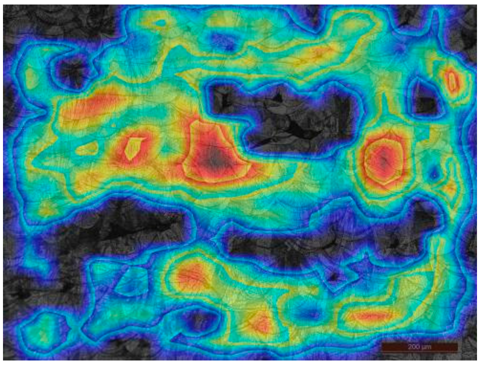

722.1

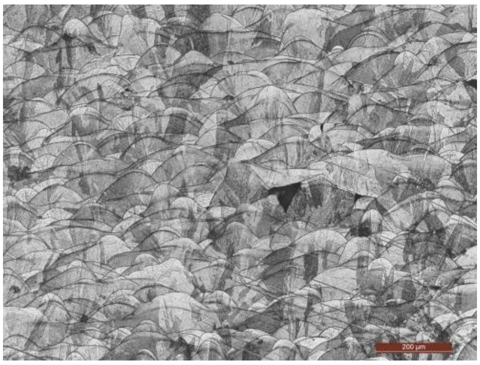

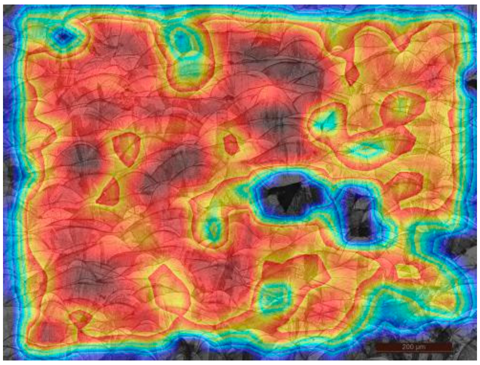

723.5

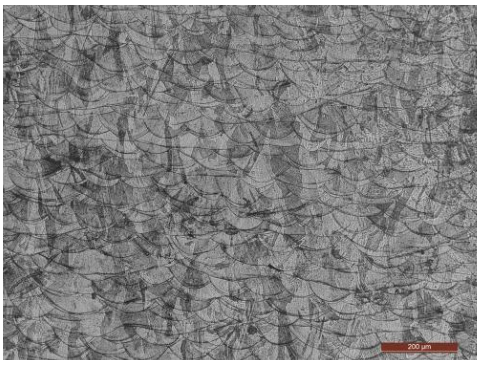

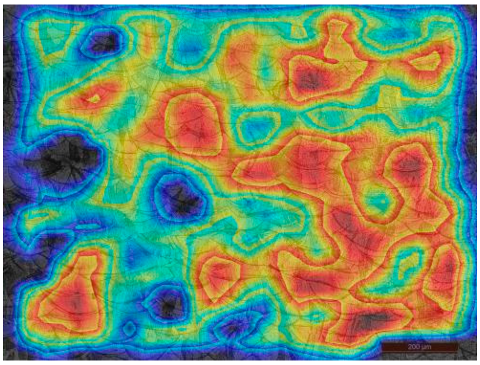

723.8

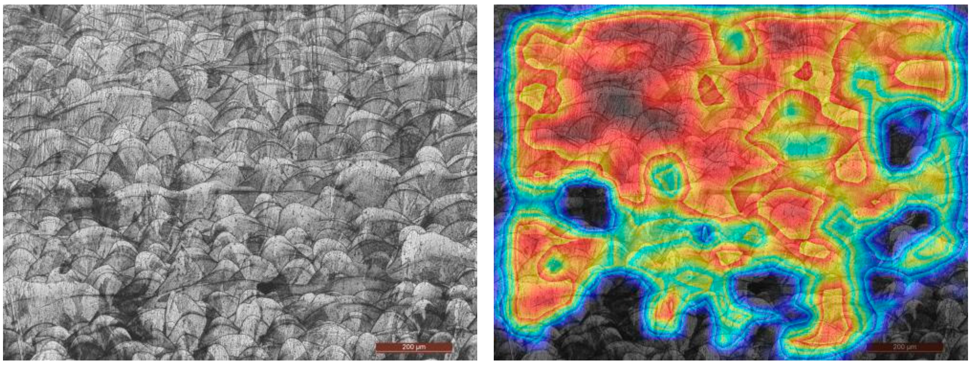

724.0

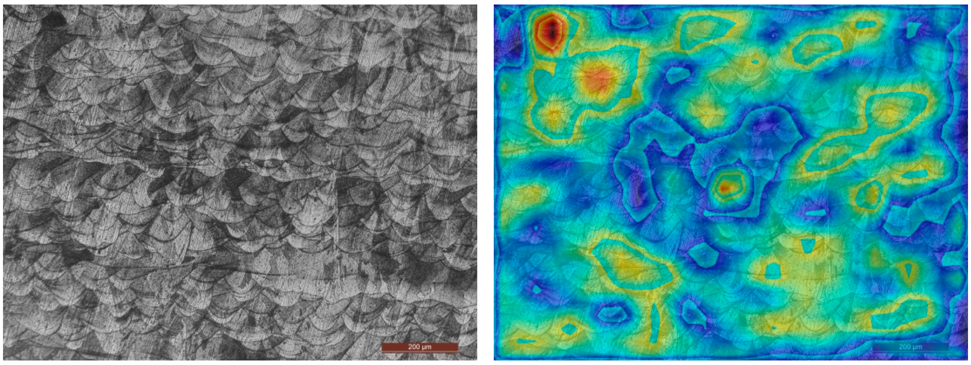

727.7

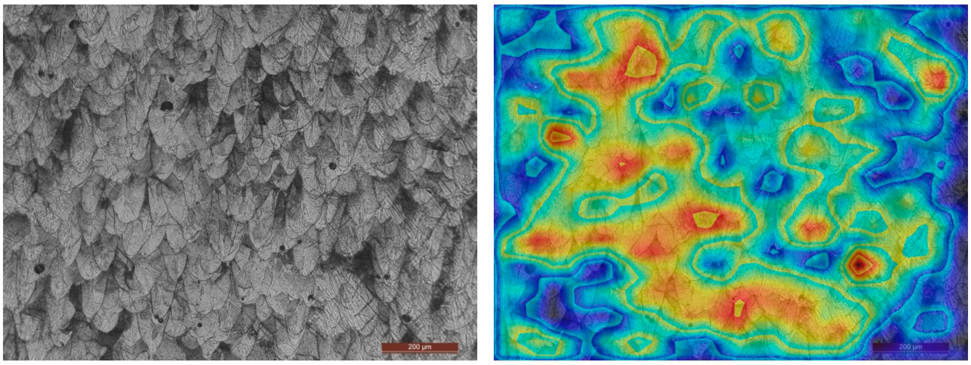

734.1

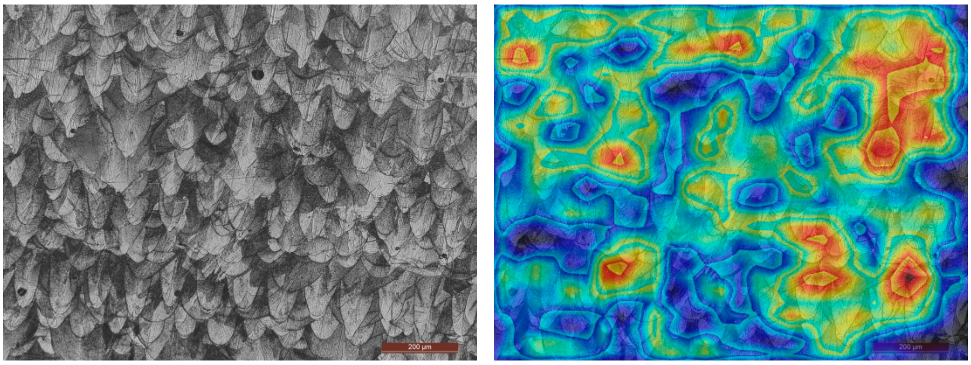

736.7

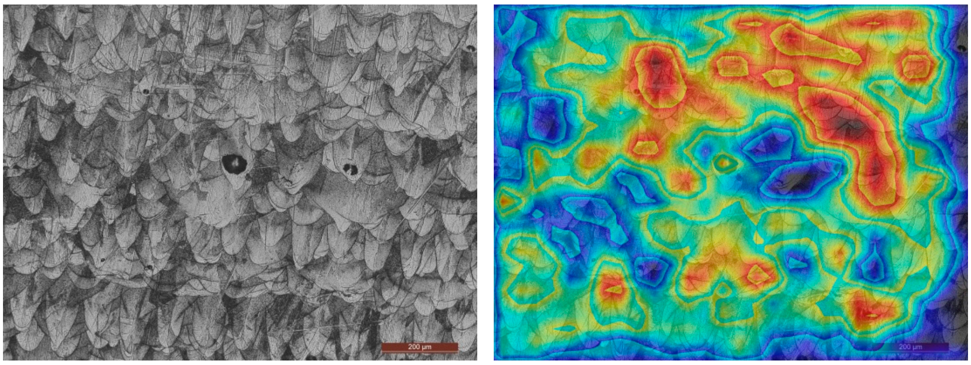

Supplementary Fig. S1 The grade class activate maps were generated by the MPR-NET with its tensile strength labels.

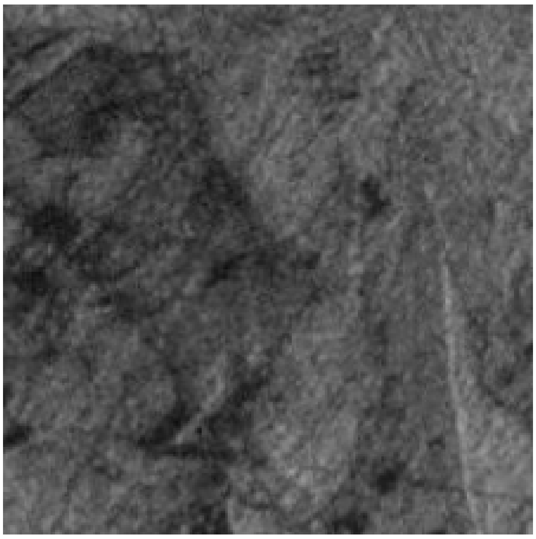

200*200

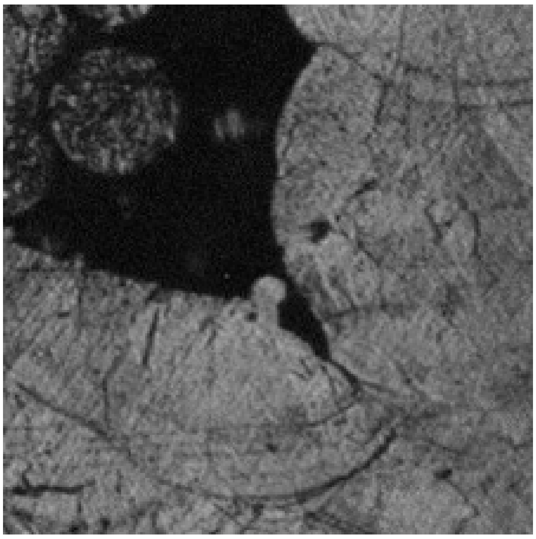

300*300

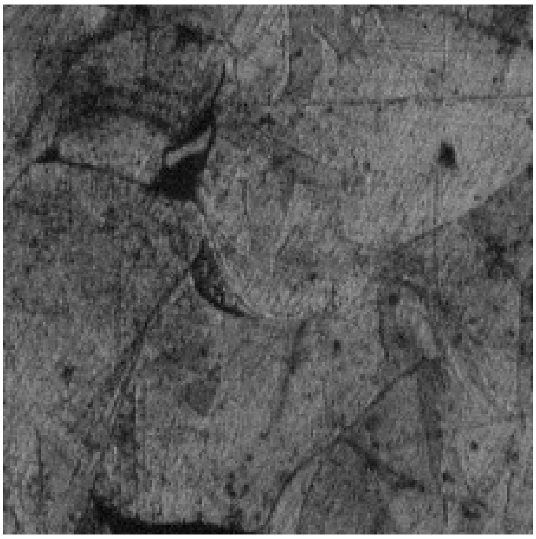

400*400

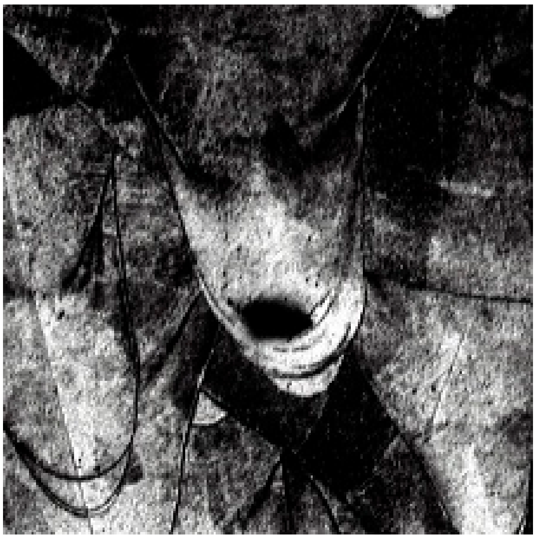

500*500

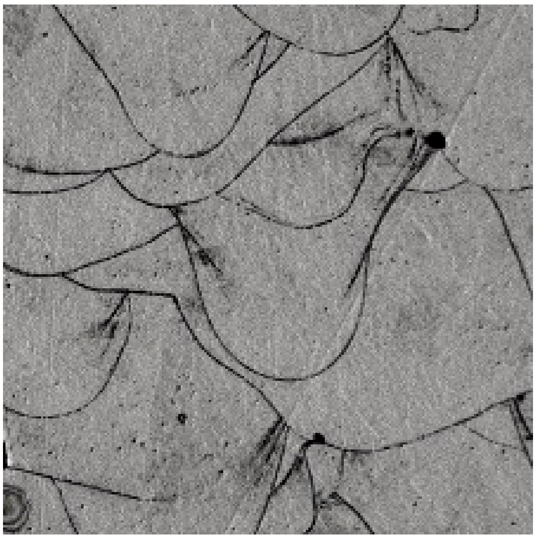

600*600

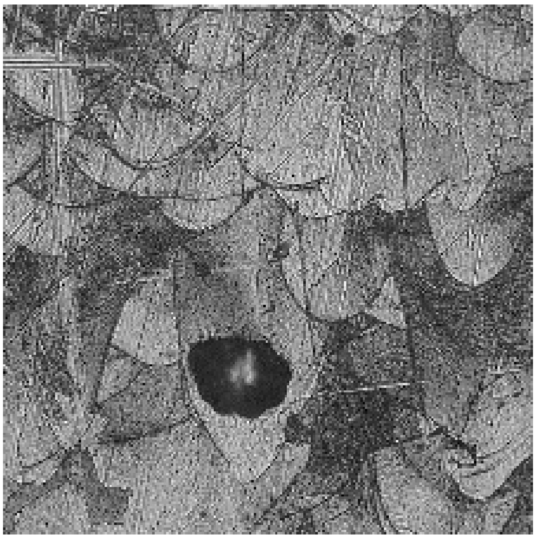

700*700

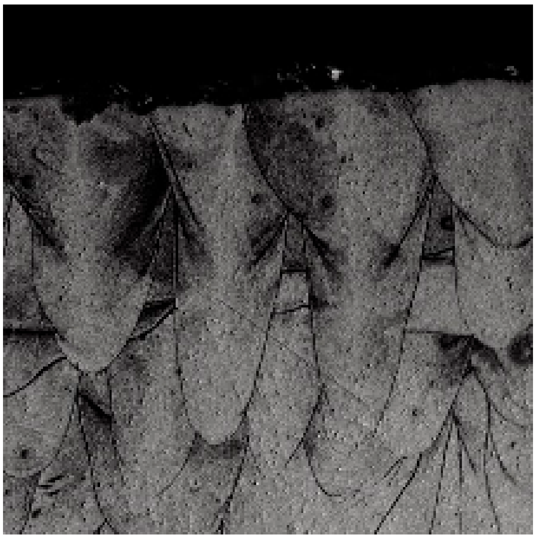

800*800

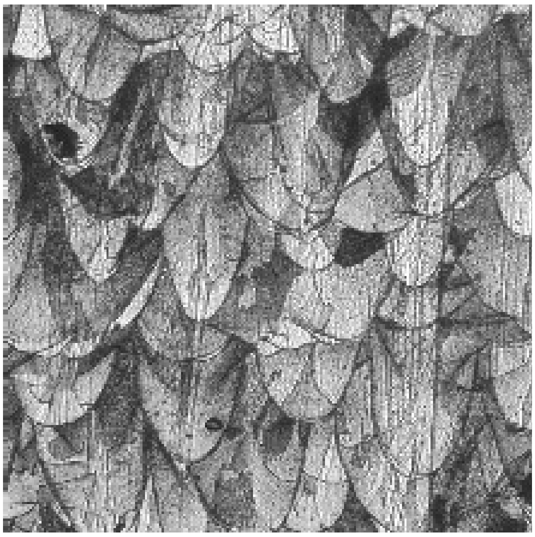

900*900

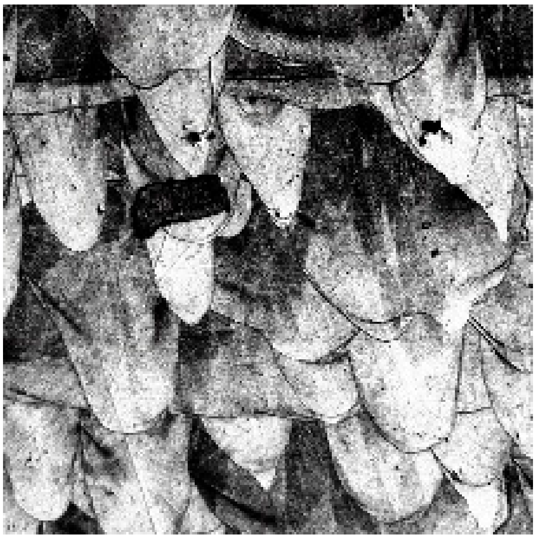

1000*1000

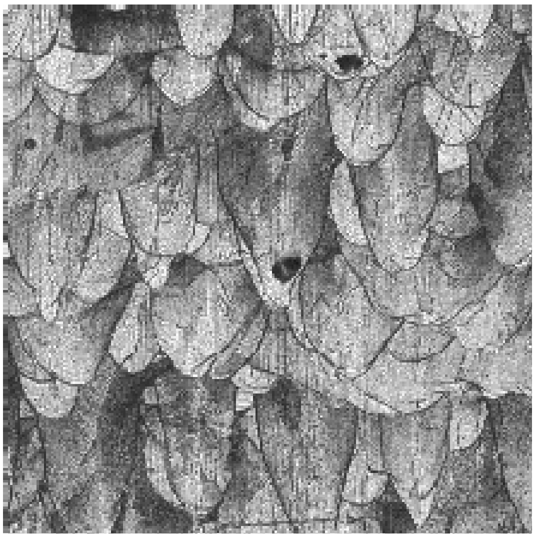

1100*1100

Supplementary Fig. S2 Metallograph images inputs with pixel dimensions ranging from 200 × 200 to 1100 × 1100 for MPR-NET.

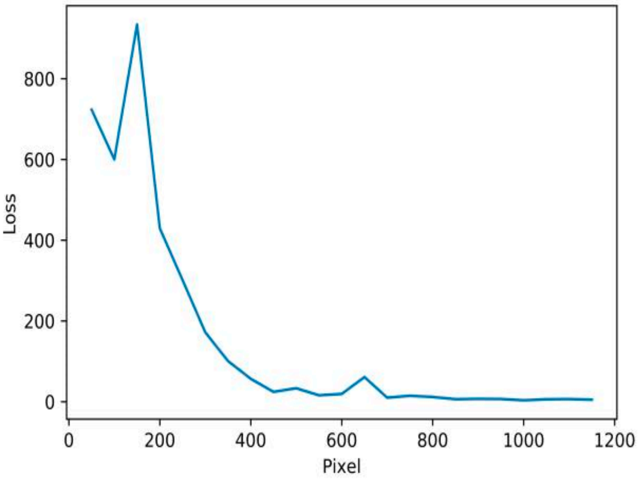

688.88 Mpa

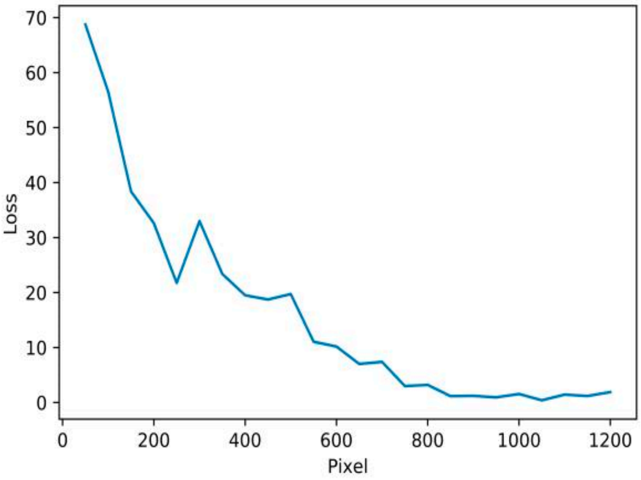

695.0 Mpa

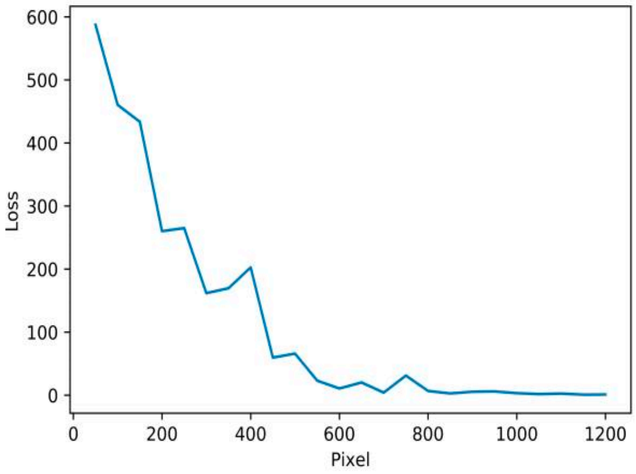

699.88 Mpa

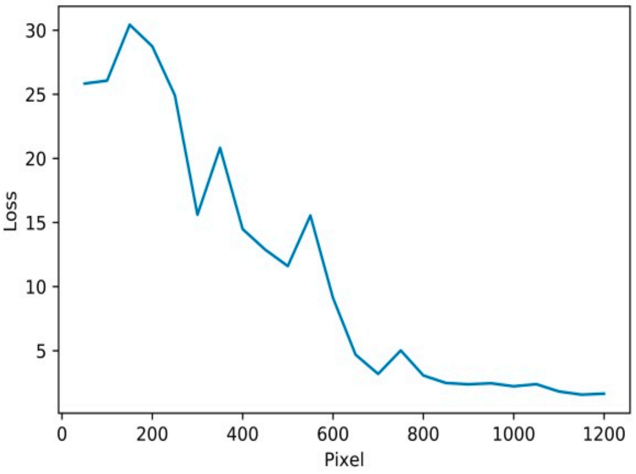

707.1 Mpa

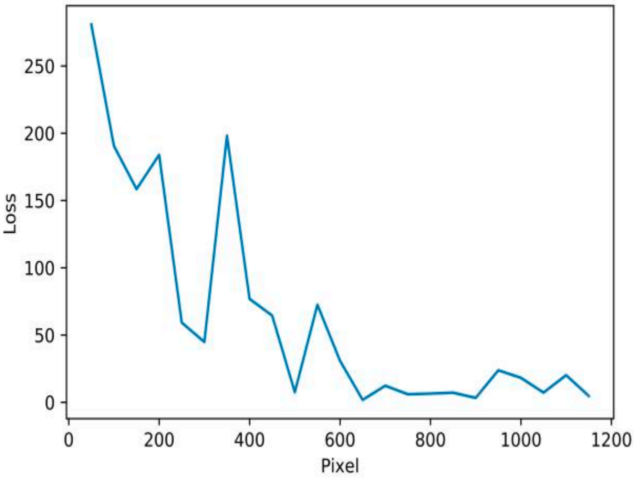

718.8 Mpa

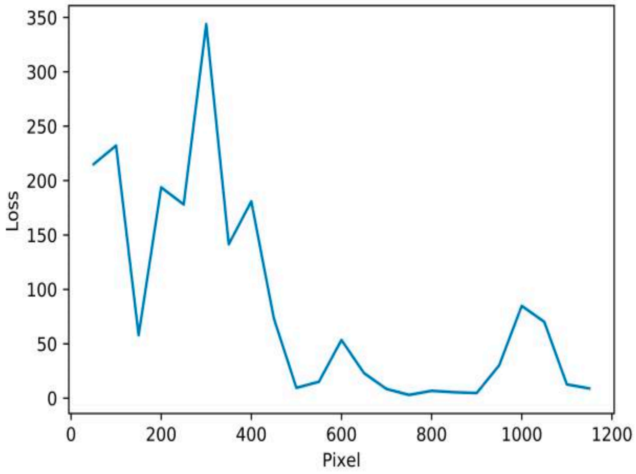

722.1 Mpa

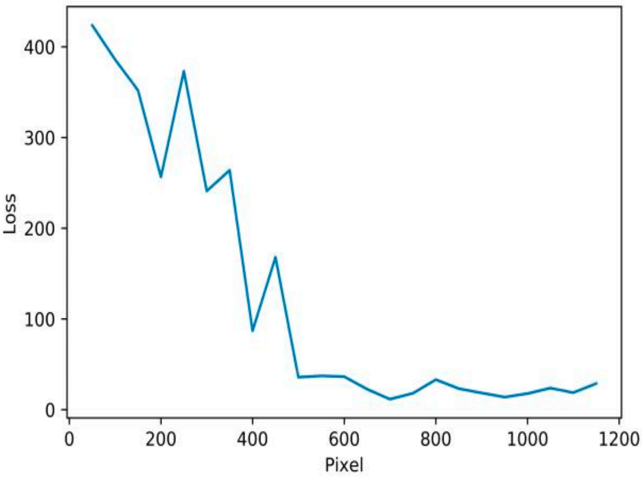

723.5 Mpa

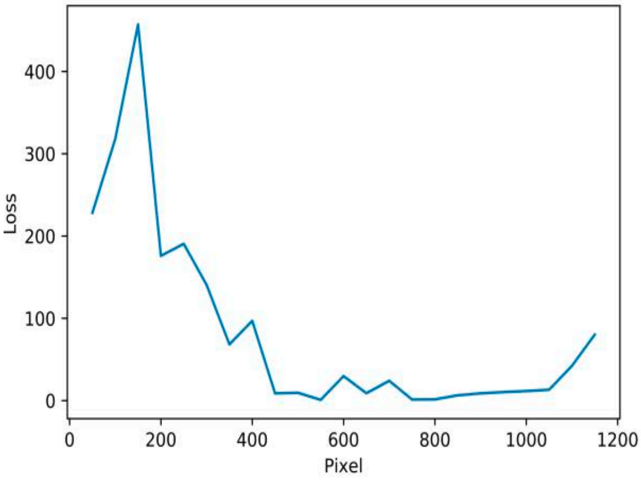

723.8 Mpa

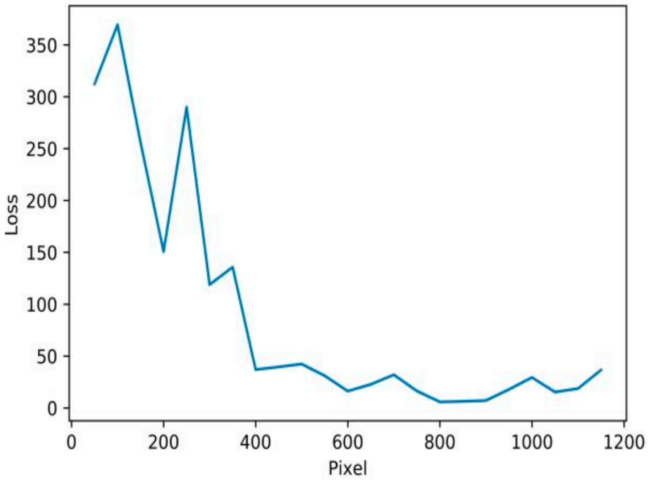

724.0 MPa

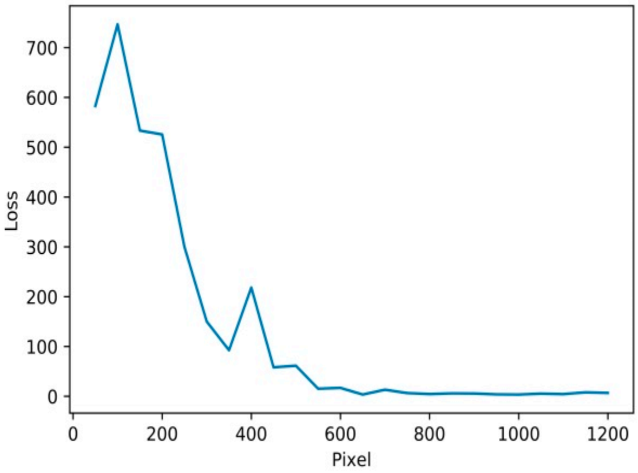

727.7 MPa

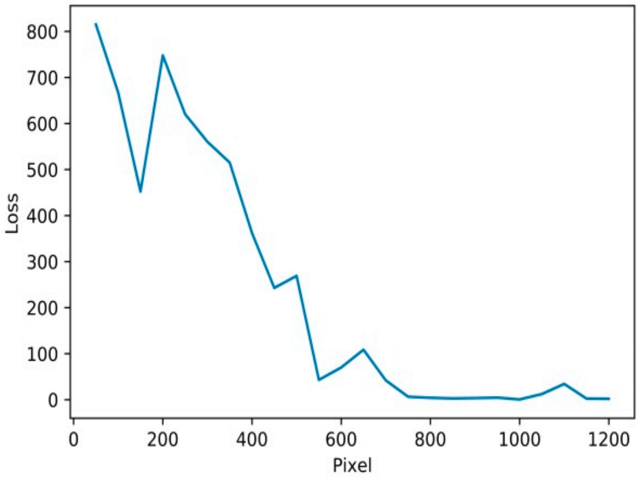

734.1 MPa

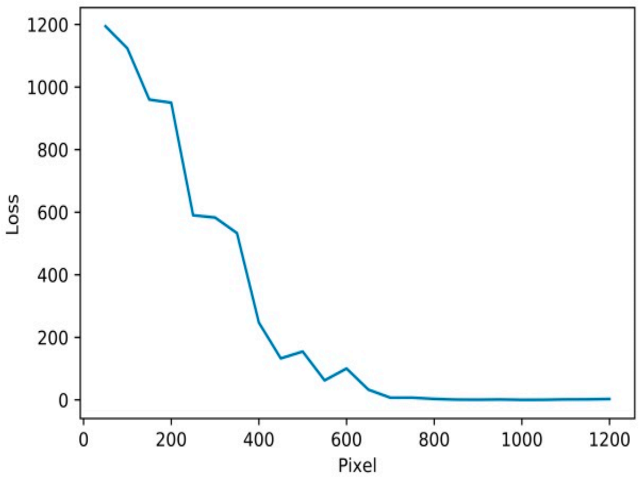

736.7 MPa

Supplementary Fig. S3 The pixel versus mean absolute error (MAE) curve of tensile strength dataset image prediction via the MPR-NET model.

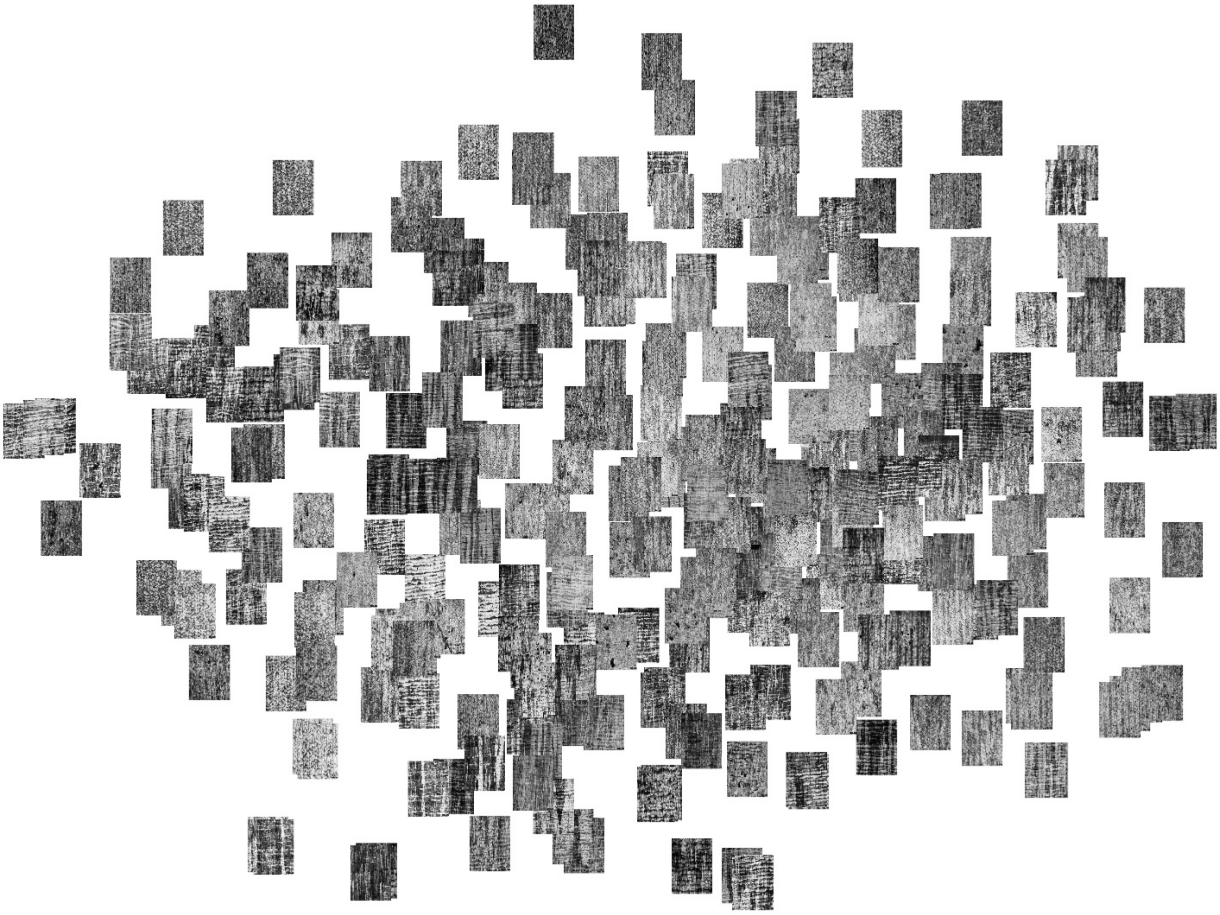

Supplementary Fig. S4 The clustering result of using t-SNE clustering on the images directly.

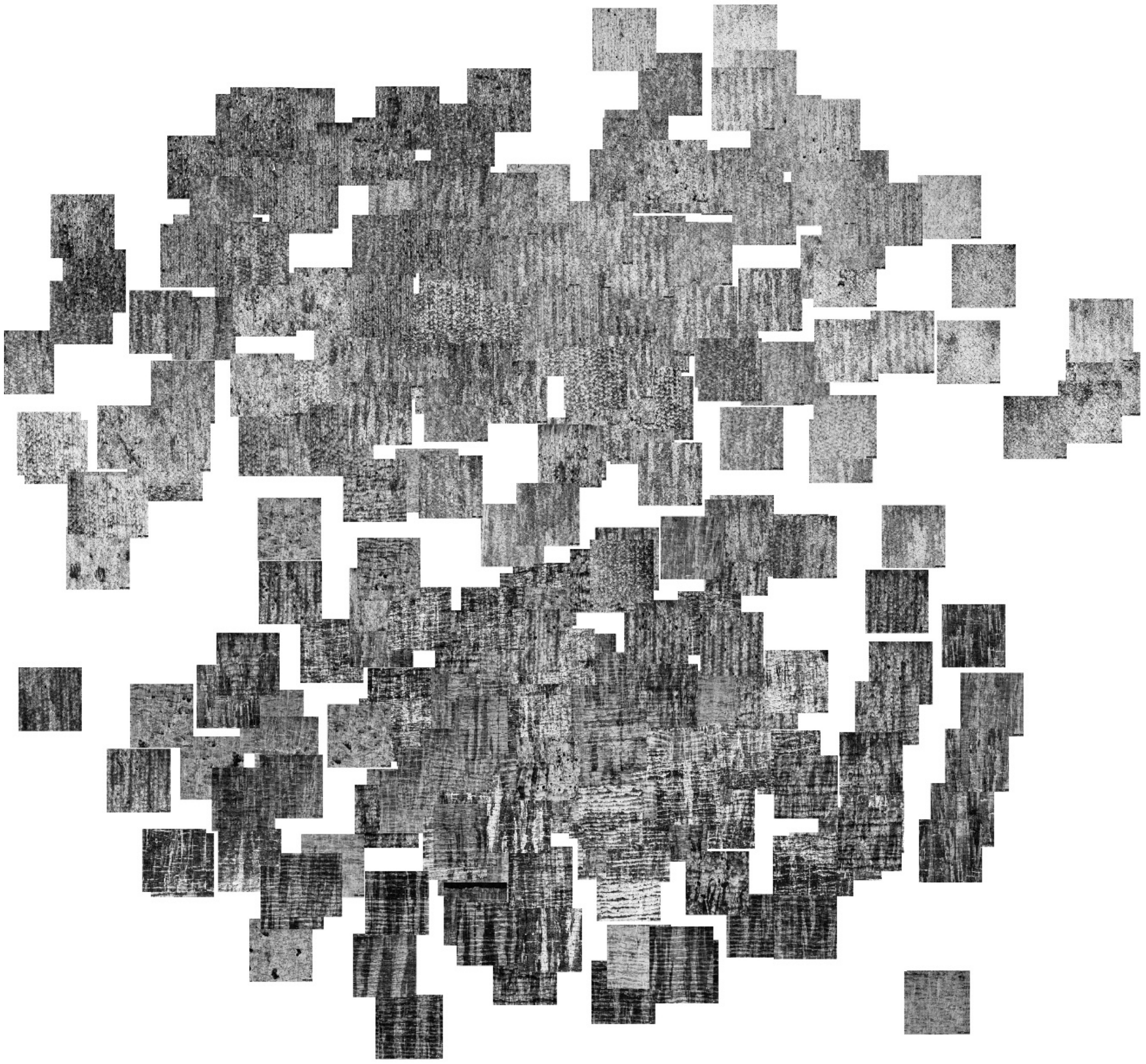

Supplementary Fig. S5 The t-SNE clustering result by feeding the tensor of the first fully connected layer of the MPR-NET.
